# Sparing of Descending Axons Rescues Interneuron Plasticity in the Lumbar Cord to Allow Adaptive Learning After Thoracic Spinal Cord Injury

**DOI:** 10.3389/fncir.2016.00011

**Published:** 2016-03-02

**Authors:** Christopher N. Hansen, Timothy D. Faw, Susan White, John A. Buford, James W. Grau, D. Michele Basso

**Affiliations:** ^1^School of Health and Rehabilitation Sciences, The Ohio State UniversityColumbus, OH, USA; ^2^Center for Brain and Spinal Cord Repair, The Ohio State UniversityColumbus, OH, USA; ^3^Neuroscience Graduate Program, The Ohio State UniversityColumbus, OH, USA; ^4^Department of Psychology, Texas A&M UniversityCollege Station, TX, USA

**Keywords:** learning, recovery, interneurons, plasticity, sparing, spinal cord injury

## Abstract

This study evaluated the role of spared axons on structural and behavioral neuroplasticity in the lumbar enlargement after a thoracic spinal cord injury (SCI). Previous work has demonstrated that recovery in the presence of spared axons after an incomplete lesion increases behavioral output after a subsequent complete spinal cord transection (TX). This suggests that spared axons direct adaptive changes in below-level neuronal networks of the lumbar cord. In response to spared fibers, we postulate that lumbar neuron networks support behavioral gains by preventing aberrant plasticity. As such, the present study measured histological and functional changes in the isolated lumbar cord after complete TX or incomplete contusion (SCI). To measure functional plasticity in the lumbar cord, we used an established instrumental learning paradigm (ILP). In this paradigm, neural circuits within isolated lumbar segments demonstrate learning by an increase in flexion duration that reduces exposure to a noxious leg shock. We employed this model using a proof-of-principle design to evaluate the role of sparing on lumbar learning and plasticity early (7 days) or late (42 days) after midthoracic SCI in a rodent model. Early after SCI or TX at 7 days, spinal learning was unattainable regardless of whether the animal recovered with or without axonal substrate. Failed learning occurred alongside measures of cell soma atrophy and aberrant dendritic spine expression within interneuron populations responsible for sensorimotor integration and learning. Alternatively, exposure of the lumbar cord to a small amount of spared axons for 6 weeks produced near-normal learning late after SCI. This coincided with greater cell soma volume and fewer aberrant dendritic spines on interneurons. Thus, an opportunity to influence activity-based learning in locomotor networks depends on spared axons limiting maladaptive plasticity. Together, this work identifies a time dependent interaction between spared axonal systems and adaptive plasticity in locomotor networks and highlights a critical window for activity-based rehabilitation.

## Introduction

Spinal cord injury (SCI) produces substantial loss of function that recovers over time (Basso et al., 1995; Curt et al., 1998; Kloos et al., 2005; Magnuson et al., 2005). At the injury site, there is typically loss of some, but not all, descending axons. Sparing of these axons contributes to functional gains over time (Basso et al., 1996; Courtine et al., 2009). While neuroplasticity of spared descending systems at the level of the lesion explains some recovery (Bareyre et al., 2004; Ballermann and Fouad, 2006; Maier and Schwab, 2006; Oudega and Perez, 2012; McKillop et al., 2013), less is known about whether these axons induce beneficial plasticity or inhibit maladaptive plasticity in spinal cord regions where they terminate caudal to the injury. In the lumbar cord, intermediate interneurons are the postsynaptic site for descending motor systems typically spared after SCI (Erulkar et al., 1966; Scheibel and Scheibel, 1969; Nyberg-Hansen, 1968; Jankowska, 1992; MacLean et al., 1995; Basso et al., 2002) and modulate reflex and locomotor function (Tillakaratne et al., 2000). Surviving fibers may produce adaptive re-wiring of these interneurons and greatly impact function. This article will systematically explore the impact of axonal sparing on downstream plasticity using a combination of behavioral and histological tools.

We evaluate the impact of spared fibers by comparing subjects that have undergone a contusion injury or complete spinal transection (TX) early after injury (7 days) vs. weeks later (42 days). To examine the possibility that surviving fibers promote recovery by fostering adaptive plasticity caudal to the injury, we employ a model of spinally-mediated instrumental learning. Past work has shown that neurons within the lumbosacral spinal cord can support learning when isolated from the brain with a thoracic (T2) transection (Liu et al., 2005; Ferguson et al., 2008; Baumbauer et al., 2009; Grau, 2014). The capacity for instrumental learning is assessed in rats by applying a nociceptive stimulus (shock) to one hind leg whenever the leg is in an extended position. Subjects given shock whenever the leg is extended (Master condition) exhibit a progressive increase in flexion duration that minimizes shock exposure. To show that this learning depends upon the relation between leg position and shock (the reinforcer), other subjects (Yoked condition) are given the same amount of shock but independent of leg position (uncontrollable stimulation). Yoked rats do not exhibit an increase in flexion duration and fail to learn when later tested with controllable stimulation, a learning impairment that has been linked to the development of nociceptive (central) sensitization (Grau et al., 1998; Crown et al., 2002; Ferguson et al., 2006, 2012; Grau, 2014). The neural plasticity supporting this learning is temporally and spatially mediated within lumbar interneuronal populations of putative central pattern generators (CPG) for locomotion (Baumbauer et al., 2009). Adaptive plasticity within interneuronal pools promotes learning and greater recovery. In contrast, learning impairments identified with this model represent maladaptive neuroplasticity in which neural changes interfere with recovery and produce functional abnormalities like central sensitization and hyperreflexia (Ferguson et al., 2012). Thus, the instrumental learning paradigm (ILP) may delineate adaptive from maladpative neuroplasticity after SCI. Here we use this paradigm to examine whether surviving fibers promote alterations in lumbosacral function and adaptive plasticity.

We reinforce our behavioral data with physiological observations derived from histological analyses of cell morphology (cell body volume, spine diameter and length) and innervation (spines per volume) in Golgi stained tissue. We hypothesized that descending fibers would lessen cell atrophy within the central gray, which should preserve cell body volume. Further, fiber loss could bring about changes in dendritic innervation that alter cellular function. On the one hand, increased spine number and spines/volume have been associated with adaptive plasticity in other neural systems (Hansen et al., 2010). However, unleashed from the brain’s tempering force, and in conjunction with alterations in cell volume, maladaptive changes in innervation could develop that undermine adaptive function.

## Materials and Methods

### Subjects

**Figure 1:**
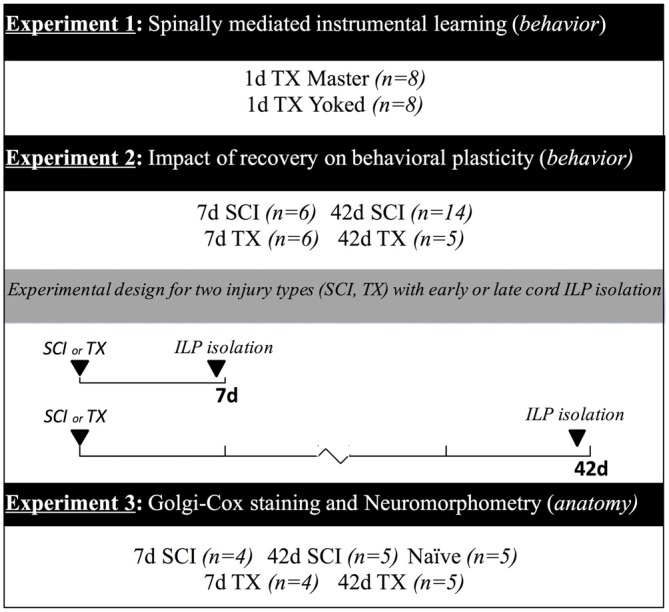
**Experimental designs.** Timeline illustrating the experimental treatments and duration of recovery. In Experiment 1, spinally transected rats were tested in the instrumental learning paradigm (ILP) with controllable (Master) or uncontrollable (Yoked) stimulation. Subjects in Experiment 2 underwent a spinal cord injury (SCI) or transection (TX) followed by instrumental testing at 7 or 42 days. In Experiment 3, rats received a SCI or transection TX and tissue was collected at 7 or 42 days. Additional subjects served as the naïve controls. The number of subjects in each condition is given in parentheses.

Adult female Sprague Dawley rats (204–290 g, Harlan, Indianapolis, IN, USA) served as subjects. Female rats were used in order to reduce the risk of bladder infection after SCI. The likelihood of this complication is increased in males and would likely confound functional outcomes in our chronic injury model. Rats were housed 2–3 per cage in a controlled environment (12 h light/dark cycle) with *ad libitum* access to food and water. All experiments were conducted under protocol approved by The Ohio State University Institutional Laboratory Animal Care and Use Committee. Figure 1 provides an overview of experimental designs and group sizes for all experiments.

### Surgical Procedures

Laminectomy and spinal cord contusions were performed as described previously (Jakeman et al., 2000; Hansen et al., 2012). Briefly, rats were anesthetized (ketamine-xylazine cocktail) and given prophylactic antibiotics (gentocin). Using aseptic techniques, an incision exposed the eighth (T8) thoracic vertebrae, and a laminectomy was performed. At T8, the vertebral column was stabilized and a severe contusion injury (250 kilodynes) applied with the Infinite Horizon device. For the transection group (TX), we opened the dura at T8 and placed a drop of lidocaine on the cord to suppress reflexes while cutting the axons with irridectomy scissors. A scalpel cut along the spinal canal confirmed that all fibers were cut. After contusion or TX, the incision was sutured and 5cc of sterile saline was given subcutaneously to prevent dehydration. Thereafter, rats received antibiotics (1 mg/kg gentocin, s.q.) and saline for 5 days, and bladders were manually expressed twice per day until bladder reflex return. Daily vitamin C pellets were given to prevent urinary tract infections (Behrmann et al., 1992). Based on prior work (Kloos et al., 2005), we conservatively estimate that there was ≤10% spared white matter after the contusion injury (Sauerbeck et al., 2013). Animals were excluded for behavioral abnormalities prior to surgery (*n* = 1), aberrant injury displacement (*n* = 1), low bodyweight (*n* = 1), or surgical complications (*n* = 1).

In experiments examining instrumental learning, a high (T2) transection was performed 24 h prior to testing to isolate the caudal spinal cord and ensure that learning was spinally mediated. After incision, the muscle and bone tissue just rostral to T2 was cleared using rongeurs exposing the cord for cauterization. The remaining gap in the cord was filled with Gelfoam (Pharmacia Corp., Kalamazoo, MI, USA) and the wound was closed with Michel clips (Fisher Scientific, Waltham, MA, USA). For additional details, see Grau et al. (1998).

### Structural Measures of Neuroplasticity

To examine how recovery affects structural indices of neuroplasticity, rats underwent a spinal transection or a contusion injury and were sacrificed 7 or 42 days later. Additional subjects served as the naïve controls. Un-perfused, fresh tissue was collected from L3–L6 and processed using a Rapid GolgiStain Kit (FD Neurotechnologies). Tissue samples had a 14-day incubation period while submerged in Golgi-Cox solution. Following rapid freezing in isopentane, tissue was cut into 200 μm coronal sections. Using brightfield microscopy (Nikon Eclipse E800) and neuron tracing software (Neurolucida, MicroBrightField), we quantified cell body volume and dendritic spines of interneurons within Lamina VI and VII by a single rater blind to group assignment (Molander et al., 1984).

Neuron location was tracked by marking the cell body within a gray matter trace constructed using Neurolucida. We selected 110 interneurons from laminae VI or VII that were fully impregnated with Golgi-Cox stain, were unobstructed by surrounding cells, and had at least one intact dendrite with clearly visible spines projecting 90 μm or more in the ventral or medial/lateral direction toward motor neuron regions. Quantification of a single dendrite per interneuron yielded 11,392 spines and represents 4–5 cells per animal. Sensitive indicators of spine dysgenesis include spine number and length (Kim et al., 2006; Hansen et al., 2010; Tan and Waxman, 2012). Atypical spine neck length and diameter indicate changes in neuronal conductance, activity thresholds, and receptor expression (Arellano et al., 2007; Araya et al., 2014). Cell volume was calculated by tracing the golgi-filled soma through progressive focal depths from top to bottom in the z-plane. Measurements of regional thickness were performed using Neurolucida at 60× magnification. Reference points were digitally placed at the top and bottom of each tissue section to measure the corresponding z-plane. This measure was used to rule out unequal tissue shrinkage effects in volumetric measurements.

Three-dimensional analysis of dendritic projections was performed using serial image stacks acquired at 100× magnification. Dendrites were traced from the cell soma to 90 μm. Bifurcating dendrites were noted and secondary branches were traced only if they projected towards ventral laminae. Dendritic spines were identified as having a clearly recognizable neck or protrusions from the dendrite with a clear indentation on either side (Kim et al., 2006). On each of the 110 dendrites, the relative position and length of spines were traced by hand using Neurolucida. Classification of spines was not performed due to the inability to reliably differentiate spine types using the Golgi method. The total number of spines was quantified to determine spine densities. In our analyses, dendritic spine number was compared relative to its cell body volume expressed as the number of spines per cubic micrometer. This measure of spines/soma volume describes the functional capacitance of the interneuron.

### Instrumental Testing

Instrumental learning was assessed 24 h following lumbar isolation (complete TX at T2). Subjects were loosely restrained in plexiglas tubes with ventilation holes at the front and slots (4 cm apart, 1.5 cm tube edge) allowing hindlimbs to hang freely above a plastic saline-filled container. A contact electrode was attached to one paw and submerged 4 mm, completing a circuit monitored by a custom script (JAB) using Spike2 Software (CED, Cambridge, UK). Circuit completion resulted in stimulus delivery to the tibialis anterior (TA) at a predetermined intensity (0.1–3.0 mA; defined by input needed to elicit a 40N ankle flexion). A flexion response to the stimulus elevated the leg, broke the circuit and terminated stimulation. Learning was evident when the contact electrode was held out of the solution to prevent shock. Data were collected in 30, 1 min duration bins and averaged per bin. Two learning measures were calculated: response duration = (time out of solution)/(response number + 1) and stimulus delivery = (time in solution exposed to stimulus). Each learning measure (response duration, stimulus delivery) of an individual subject was averaged across the 30 testing bins to yield a single score from which group means were calculated.

### Open Field Locomotor Outcomes

Open field locomotor recovery was assessed using the Basso, Beattie, Bresnahan (BBB) Locomotor Rating Scale (Basso et al., 1995). Scores range from no hindlimb movement (0) to normal locomotor function (21). Categories including joint movement, weight support, plantar stepping, toe clearance, trunk, and tail control were considered for scoring. Two raters blind to group assignment examined the open field activity of each rat for 4 min.

### Statistics

The data were analyzed using an analysis of variance (ANOVA) or analysis of covariance (ANCOVA) using SuperAnova Software (now SAS). *Post hoc* comparisons were performed using Duncan’s New Multiple Range Test. Multivariate analysis of variance (MANOVA) were run *post hoc* to compare Experiment 1 and Experiment 2. In all cases, a criterion of *p* < 0.05 was used to judge statistical significance.

## Results

### Experiment 1: Instrumental Learning in Transected Female Rats

**Figure 2:**
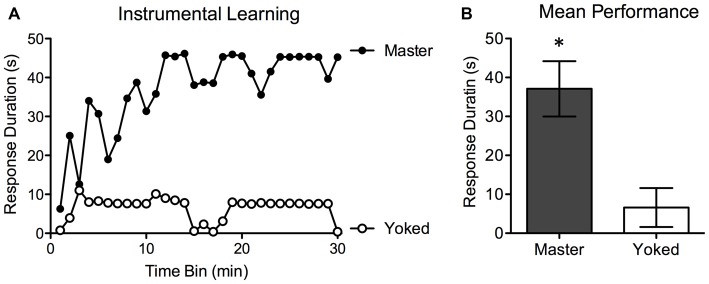
**Experiment 1.** Mean instrumental learning curves for the Master and Yoked groups **(A)**. Controllable stimulation (Master) induced a progressive increase in response duration as a function of training in spinally-transected rats **(A)**. Subjects that received the same amount of stimulation independent of leg position (Yoked) failed to learn. Graph A depicts group averages for each 60 s bin over the 30 min testing period. Mean performance over the entire 30 min time period (+ the SEM) is depicted in **(B)**. *p < 0.05.

Prior to testing, shock intensity was adjusted to elicit an equally strong flexion response. There were no differences in the shock intensity required to elicit a response, *F*_(1,14)_ < 1.0, *p* > 0.05. Test performance for Master (*n* = 8) and Yoked (*n* = 8) rats is depicted in Figure 2. Rats in the Master condition exhibited a progressive increase in flexion duration that minimized net shock exposure (mean response duration = 37.05 ± 6.04 s). This learning was not observed in yoked subjects (mean response duration: 6.51 ± 4.21 s). An ANOVA confirmed that overall group differences were statistically significant, *F*_(1,14)_ = 17.04, *p* < 0.001. As in prior work, trend analyses showed that the change in response duration observed over time was predominantly linear, *F*_(1,406)_ = 22.08, *p* < 0.001. Because Master and Yoked rats received the same amount of stimulation (by design), there were no group differences in net shock exposure. Our results (Figure 2) replicate past studies and demonstrate spinally-mediated instrumental learning in female rats. Next, we used this behavioral task to explore adaptive capacity 7–42 days after injury.

### Experiment 2: Spared Fibers Restore Adaptive Plasticity After Spinal Injury

Subjects had T8 spinal contusion (SCI; *n* = 20) or transection (TX; *n* = 11) and instrumental testing was performed 7 or 42 days later. To assure that learning in the SCI group was spinally mediated, the caudal spinal cord was isolated from brain and cervical inputs a day before testing. To control for the effect of surgery, all subjects received the same surgery to isolate the cord. Instrumental testing was performed as described above. In addition, both locomotor performance and mechanical reactivity were assessed prior to the T2 transection and before instrumental testing.

**Figure 3:**
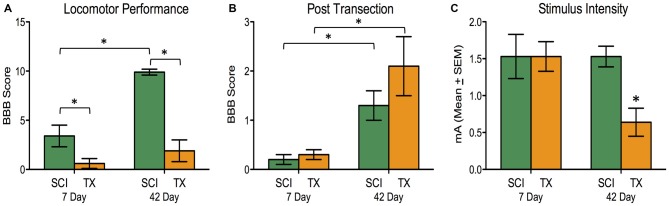
**Experiment 2.** A SCI led to superior locomotor performance that improved over the recovery period compared to complete TX **(A)**. A subsequent spinal transection at T2 showed greater hindlimb motor actions after 42 days vs. 7 days for both SCI and TX groups. The SCI and TX groups did not differ from each other at either timepoint (**B**; note the change in scale compared to **A**). Significantly lower stimulation intensity of the tibialis anterior (TA) elicited flexor withdrawal for the 42 day TX group **(C)**. *p < 0.05.

The SCI group demonstrated superior locomotor performance compared to TX groups (Figure 3A). The magnitude of this difference increased over time. An ANOVA confirmed that days of recovery, injury type, and their interaction, were statistically significant, all *F*’s > 12.63, *p* < 0.005 (Figure 3A). After ILP isolation of the cord (T2 transection), both SCI and TX groups showed improved BBB scores at 42 days compared to the 7 day group, *F*_(1,27)_ = 17.24, *p* < 0.001 (Figure 3B). This was confirmed with *post hoc* comparisons (*p* < 0.05). No other group comparisons were significant (*p* > 0.05).

Hindlimb flexion reactivity was measured using the stimulus intensity required to elicit a 40N flexion force following spinal isolation. Transected rats given 42 days to recover were more responsive to the test shock and required less stimulation (Figure 3C). An ANOVA showed that the effect of injury, days to recover, and their interaction, approached significance, all *F*’s > 3.84, *p* < 0.061. This yielded a main effect of injury group, *F*_(3,27)_ = 3.26, *p* < 0.05. *Post hoc* comparisons showed that the TX group at 42 days differed from the other groups (*p* < 0.05). No other group comparisons were significant (*p* > 0.05).

**Figure 4:**
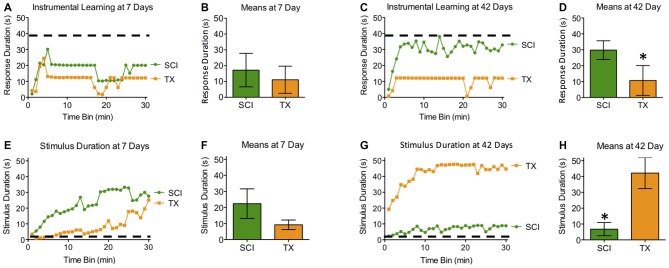
**Experiment 2.** At 7 days after injury, both SCI and TX treated groups exhibited poor learning that suggests the development of maladaptive plasticity **(A,B)**. Both groups received comparable levels of stimulation **(E,F)**. **(A,E)** depict group averages for each 60 s bin over the 30 min testing period. Group means (+ SEM) derived from individual scores over the entire 30 min testing period are depicted in **(B)** and **(D)**. Only rats that recovered for 42 days in the presence of spared fibers (SCI) exhibited superior learning **(C,D)**. The 42 day TX group exhibited poor learning shown by significantly lower response duration **(C,D)** and significantly more shock exposure compared to the SCI group **(G,H)**. Error bars indicate the standard error of the mean. *p < 0.05.

Subjects tested 7 days after a SCI or TX exhibited poor learning (Figures 4A,B,E,F). When tested 42 days after injury, only the SCI group exhibited learning that was comparable to that observed in naïve rats with acute ILP isolation tested with controllable stimulation (Master). While the main effects of injury and recovery period were not statistically significant, both *F*’s < 1.65, *p* > 0.05, the linear components of the Time × Injury and Time × Recovery Period interactions were significant, both *F*’s > 8.65, *p* < 0.005 (Figures 4C,D,G,H). To further explore the nature of this interaction, we examined the linear component of the time × injury interaction at 7 and 42 days. These analyses showed that the SCI and TX groups differed at 42 days, *F*_(1,493)_ = 4.58, *p* < 0.05, but not at 7 days, *F*_(1,290)_ = 2.43, *p* > 0.05.

Spinally transected rats that failed to learn 42 days after injury also received more shock, yielding a significant injury × recovery period interaction, *F*_(1,27)_ = 12.80, *p* < 0.005. Reasoning that this effect could be related to differences in shock reactivity prior to testing, we performed an ANCOVA with shock intensity as a covariate. Shock intensity did account for a significant proportion of the variance, *F*_(1,26)_ = 11.92, *p* < 0.005. However, a significant injury × recovery period interaction remained, *F*_(1,26)_ = 7.56, *p* < 0.05. *Post hoc* comparisons showed that rats tested 42 days after a spinal transection received greater stimulation than both the SCI treated group tested at 42 days and the transected group tested at 7 days (*p* < 0.05). No other group differences were statistically significant (*p* > 0.05).

To describe learning relative to normal, we ran individual *post hoc* analyses comparing SCI and TX rats to the Master rats in Experiment 1. We postulated that the isolated cord at 24 h would be the best representation of normal lumbosacral plasticity before injury mechanisms ensue. As such, we used a Master controlled MANOVA against groups that recovered with or without sparing. Response duration was significantly lower than Master controls for all groups except the 42 day groups (*p* < 0.05). Stimulus delivery was significantly higher than Master controls only for rats that recovered to 42 day TX rats (*p* < 0.05).

### Experiment 3: Spared Fibers Rescue Injury-Induced Changes in Cell Morphology

**Figure 5:**
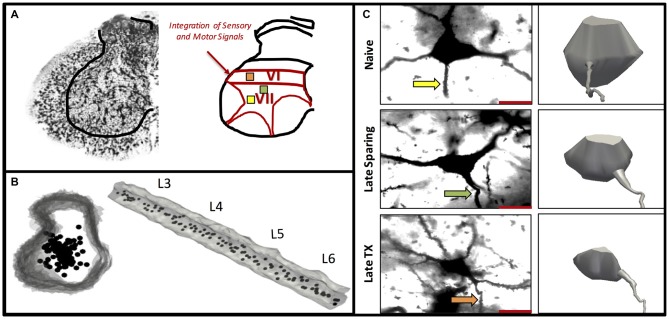
**Experiment 3.** A representative Golgi-stained lumbar section illustrates intermediate laminae VI–VII where integrator neurons were selected **(A)**. Three-dimensional rendering of the lumbar spinal cord L3–L6 shown cross sectionally and longitudinally **(B)**. The location and distribution of interneurons included in the analyses were equally distributed along the rostral-caudal axis within intermediate lamine **(B)**. A representative Golgi-stained interneuron cell body from Naïve, 42 day SCI, and 42 day TX groups along with three-dimensional cell body reconstruction illustrates interneuronal atrophy that occurs primarily following TX **(C)**. The arrows indicate the dendrite selected for spine analysis **(C)**.

**Figure 6:**
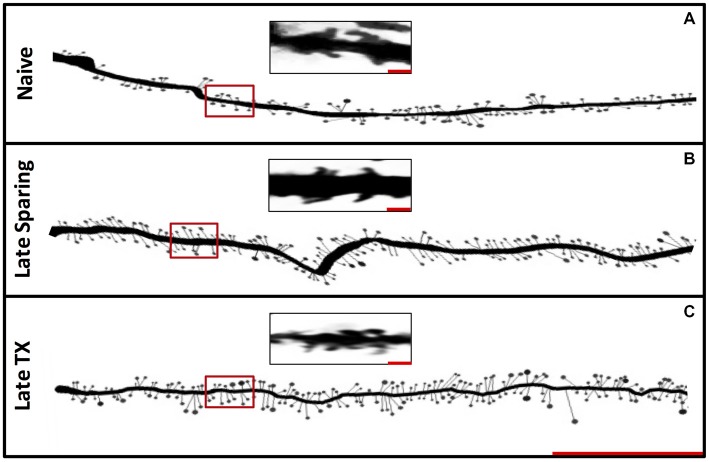
**Experiment 3.** Representative Golgi-stained dendrites from Naïve, Late SCI and Late TX groups **(A)**. The distribution of spines along 90 μm illustrates a trend toward increased spine density in both SCI and TX groups at late time points (**B**; Scale bar = 30 μm). Magnified images of dendritic spines from locations denoted by the red boxes within the first 30 μm (**C**; Scale bar = 1 μm).

**Figure 7:**
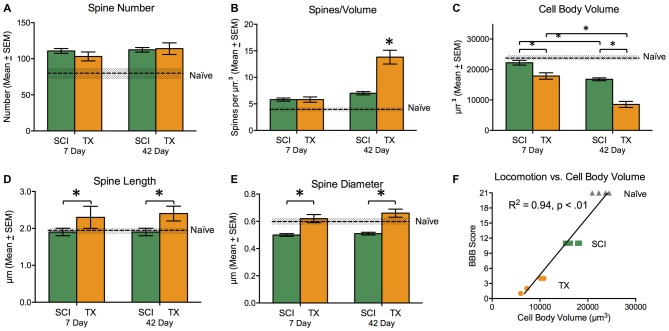
**Experiment 3.** Measures of cell morphology 7 and 42 days after SCI (green) or TX (orange). Data from the Naïve controls is illustrated by the dashed line. Error bars indicate the standard error of the mean. SCI generally increased spine number **(A)**. In the absence of descending fibers (TX), there was a dramatic increase in spines/volume that increased over days **(B)**. A spinal transection also led to a reduction in cell body volume and this effect was mitigated by the presence of spared fibers (SCI). **(C)** Injury type had opposite effects on spine length, with a reduction after SCI and an increase after TX **(D)**. There was a reduction in spine diameter after SCI **(E)**. Behavioral recovery at day 42 was highly correlated with cell body volume **(F)**. *p < 0.05.

Subjects had T8 spinal contusion (SCI; *n* = 9) or transection (TX; *n* = 9) and tissue collection occurred 7 or 42 days later alongside naïve controls (*n* = 5). Subjects in the SCI groups exhibited superior locomotor performance that improved over the recovery period. An ANOVA confirmed that BBB scores depended upon both injury type and days of recovery, *F*_(1,13)_ = 7.96, *p* < 0.05. For golgi analysis in these rats, the spinal cord region that was sampled is illustrated in Figures 5A,B. Measures of cell morphology revealed alterations in both the spines and interneuronal volume after injury (Figures 6, 7C; Naïve = 22739.7 μm^3^ ± 618; 7 days SCI = 22221.7 μm^3^ ± 902; 7 days TX = 17871.8 μm^3^ ± 1206; 42 days SCI = 16508.3 μm^3^ ± 748; 42 days TX = 8144.5 μm^3^ ± 1030). Both the complete loss of descending fibers (TX) and a contusion injury (SCI) led to an increase in spine number relative to the naïve controls (Figure 7A; Naïve = 84.0 ± 9.4, 7 days SCI = 110.8 ± 3.9, 7 days TX = 103.2 ± 7.2; 42 days SCI = 112.4 ± 3.6; 42 days TX = 114.0 ± 9.4). We found that SCI and TX yielded a similar increase in spine number at 7 and 42 days, all *F*’s < 1.0, *p* > 0.05 (Tracings in Figure 6, quantification in Figure 7).

In the absence of spared fibers (TX), there was dramatic increase in spines per volume (Figure 7B), and this effect grew over days. Relative to naïve subjects, SCI led to an increase in spines/volume, *F*_(2,11)_ = 17.5, *p* < 0.001. *Post hoc* comparisons revealed that early and late SCI treated groups differed from the naïve group (*p* < 0.05). In addition, SCI rats given 42 days to recover exhibited a significant (*p* < 0.05) increase in spines/volume relative to the 7 day SCI group. A comparison of SCI groups to the TX treated rats revealed that the spines/volume depended upon both injury and days of recovery, *F*_(1,13)_ = 13.19, *p* < 0.005 (Figure 7B). For these comparisons, the predominant group difference was between the 42 day TX group, which differed from both SCI groups and the 7 day TX group (*p* < 0.05).

A spinal transection led to a significant decrease in cell body volume (Figures 5C, 7C) and this effect was mitigated by the presence of spared fibers in the SCI group. A comparison of the SCI treated groups to the naïve controls showed that injury had a significant effect, *F*_(2,11)_ = 25.4, *p* < 0.0001. *Post hoc* comparisons showed that the SCI treated rats evaluated 42 days after injury differed from the other two groups (*p* < 0.05). No other group differences were significant. Likewise, a comparison of the SCI and TX conditions revealed a significant effect of both injury type and days of recovery, both *F*’s > 44.72, *p* < 0.0001. *Post hoc* comparisons showed that all group differences were significant (*p* < 0.05) with the exception of the 7 day TX and 42 day SCI conditions (*p* > 0.05).

Relative to the naïve controls, SCI did not have a significant effect on spine length (Figure 7D), *F*_(2,11)_ = 1.26, *p* > 0.05. A comparison of SCI and TX treated groups revealed a significant effect of injury type, *F*_(1,13)_ = 6.53, *p* < 0.05, stemming from the greater length observed after spinal transection. Spine diameter (Figure 7E) was reduced in SCI treated rats relative to the naïve controls, *F*_(2,11)_ = 5.43, *p* < 0.05. *Post hoc* comparisons showed that the two SCI treated groups differed from the naïve group (*p* < 0.05). Relative to SCI treated rats, a spinal transection produced an increase in spine diameter, *F*_(1,13)_ = 38.00, *p* < 0.0001. *Post hoc* comparisons confirmed that the two transected groups differed from the SCI treated groups (*p* < 0.05).

Finally, we examined the relationship between cell body volume and behavioral recovery (Figure 7F). The analysis revealed a strong relationship between interneuronal cell body volume and behavioral recovery (*r* = 0.97, *p* < 0.01). Our results indicate that spinal injury induces alterations in cell morphology, including an increase in spines/volume and a decrease in cell body volume. These effects were stronger after a spinal transection, suggesting that descending fibers mitigate injury-induced alterations in cellular function.

## Discussion

Our study documents novel behavioral and structural indices of plasticity within the lumbar cord after thoracic SCI. We found that interneurons known to critically regulate locomotor pattern generation develop a maladaptive, hyperexcitable phenotype in the absence of descending axonal communication during recovery. However, long-term exposure to spared axons prevented a maladaptive phenotype while restoring segmental learning behavior. We show that at 7 days after SCI, adaptive, sparing-induced structural or behavioral plasticity was diminished. Late after SCI, the lumbar cord demonstrated near-normal learning behavior while interneurons maintained cell body volume and had lower dendritic spine densities. Together, our work demonstrates the remarkable ability of spared axons to generate plasticity in remote locomotor neuron networks and promote functional gains. These findings identify important anatomical targets whereby afferent cues provided via rehabilitation may improve below-level functional deficits.

**Figure 8:**
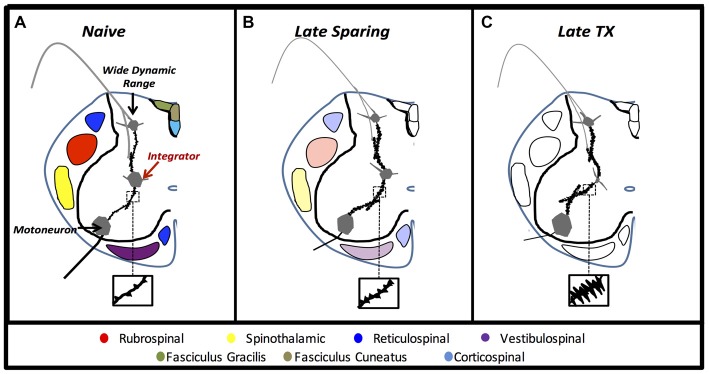
**Experiment 3.** Anatomical cartoons demonstrate structural plasticity within pre-motor laminae VII interneurons in rats that recover to late time points with no injury **(A)**, with some axonal sparing **(B)**, or without axonal sparing **(C)**.

Findings of cell-soma atrophy in laminae VII interneuron populations highlight a critical region of the presumptive CPG that is compromised by upstream cord injury. Reductions in neuronal soma volume indicate fatigue, cell stress, and even impending apoptosis in a variety of models (Sofroniew et al., 1986; Ross and Ebner, 1990; Charles et al., 1996). Further investigation is needed to determine if the *in vivo* somal atrophy we report here reflects stress, fatigue or apoptotic processes after SCI. Nevertheless, the marked atrophy of interneurons well caudal to the injury suggests that behavioral impairments are due at least in part to downstream maladaptive plasticity. Indeed, Figure 8 depicts the structural neural plasticity influenced by axonal sparing within putative CPGs. Laminae VII interneurons within the intermediate gray matter of the lumbar cord integrate phasic afferent signals with supraspinal motor input to convey synaptic drive to motor neuron pools during walking (Jankowska, 1992; MacLean et al., 1995). Our findings of structural plasticity in these neuron networks extend previous electrophysiological findings that indicate maladaptive and hyperexcitable network communication after SCI (Rank et al., 2015). In response to lost supraspinal drive, dendritic outgrowth may reflect new connections that require greater metabolic or trophic support that is scavenged from the soma. Similar dendritic findings on alpha-motoneurons indicated spasticity and hyperreflexia (Bandaru et al., 2015). It appears that spared axons protect remote neuron networks from developing hyperexcitable communication. We suspect that maladaptive plasticity observed within laminae VII interneurons may be a central component to below level functional deficits such as pain, spasticity, and locomotion.

It is well established that axonal sparing facilitates motor capacity in lumbar segments after SCI (Basso et al., 1996, 2002; Wang et al., 1998; Cohen-Adad et al., 2014). In both rat and feline models, recovery from incomplete SCI followed by complete transection augments motor activity from the lumbar cord (Basso et al., 1996; Cohen-Adad et al., 2014). The spinal-centric mechanisms involved in this effect, however, have been less clear. As such, we employed the ILP to obtain a sensitive behavioral measure of interneuron plasticity in lumbar segments (L4-S2; Liu et al., 2005). We found that even a small complement of axons (estimated <10%; Sauerbeck et al., 2013) can generate plasticity in the lumbar enlargement that facilitates learning. This plasticity required several weeks of axon exposure during a period of spontaneous recovery suggesting that sparing does more than simply preserve below-level neuronal function. In contrast, the absence of descending axons produced marked atrophy and aberrant spine formations of the lumbar intermediate interneurons within the first week and worsened over 6 weeks. The interneuron phenotypes observed in intermediate gray matter indicated hyperexcitability and an increase in inappropriate connections (Hains and Waxman, 2006; Kim et al., 2006; Rank et al., 2015). Together, our work suggests that descending fibers preserve physiological function by promoting adaptive structural changes in lumbar neurons that allow learning.

The failure of spinal learning early after SCI is not surprising. By 7 days after thoracic SCI, spinal shock accompanies pronounced cellular and molecular events in the lumbar cord that may jeopardize neuronal plasticity (DiTunno et al., 2004; Finger et al., 2004; Gomez-Pinilla et al., 2012; Gwak et al., 2012). Both increases in inflammatory activities and declines in trophic production identify a toxic milieu for proper neurotransmission (Hutchinson et al., 2004; Gomez-Pinilla et al., 2012; Hansen et al., 2013). Indeed, such events are predictive of the development of below-level afferent hypersensitivity and prevent activity-based motor relearning (Detloff et al., 2008; Smith et al., 2009; Hansen et al., 2013). Thus, it may be possible that a toxic microenvironment overrides any beneficial input from spared axons early after SCI in our model. It has long been known that trophic support and inflammatory actions are critical regulators of neuronal phenotype (Yirmiya and Goshen, 2011). Increases in cytokine expression and declines in trophic factors alter dendritic spine expression, produce apoptosis and interfere with metabolic patterns (Yirmiya and Goshen, 2011). After SCI, the resolution of these factors overtime likely permit adaptive changes at the level of the synapse. To this end, we clearly show the emergence of adaptive structural plasticity within neuronal dendrites alongside considerable learning late after SCI. Thus, the timing of these events identifies an important interaction with the activities of spared axons that provide a novel opportunity for targeted intervention. We suggest that the capacity to integrate descending motor and afferent sensory signals in the lumbar cord depends on time, which is a determining factor for recovery after SCI.

Task-specific neurorehabilitation may enhance sparing-induced plasticity and facilitate learned-reflex modulation within interneuron networks during locomotion. Even in a complete transect model, different training tasks prevent spinal learning on the opposite limb (Bigbee et al., 2007). Thus, it may be possible to use environmental cues to modulate maladaptive plasticity and improve learning. This theory is promising in cases of spasticity, which is clinically observed in both complete and incomplete injuries. Delivery of afferent signals facilitates sensorimotor integration in the lumbar cord and reduces reflex gains during locomotion. This strategy has been successful in humans when H-reflex training is shown to improve walking patterns (Thompson et al., 2013). More work is needed to determine the complexities of spinal cord plasticity after incomplete SCI and the ability to preferentially target sensorimotor interneuron populations in the lumbar cord.

Our work identifies a time-dependent interaction between spared axonal relays and Lamina VII interneurons responsible for phasic sensory input and motor output. Exposure to spared axons prevents neuronal atrophy and restores learning that is lost early after SCI. Therapeutic strategies that consider timing and facilitate the integration between spared axonal relays and pattern generator networks may potentiate motor relearning and recovery. Future studies will determine if task specific training can facilitate the integration between spared and segmental neuronal signals to improve recovery and reflex gains during locomotion.

## Author Contributions

DMB and CNH were primarily responsible for designing the experiments with JWG also contributing substantially to experimental design for the ILP studies. SW and JWG designed the statistical analysis plan for the studies that was implemented by DMB and CNH. DMB, CNH and TDF designed the golgi experiments including methods, analysis and interpretation. JAB developed the data collection system for ILP, supervised and trained CNH in data acquisition. JWG, JAB, CNH, SW and DMB all contributed equally to data interpretation for ILP. All authors drafted the manuscript, gave final approval for the version submitted for review and agree to be accountable for the accuracy and integrity of the studies reported here.

## Funding

This work is funded by NIH grants: 1F31NS080512-01 (CH), 1RO1NS074882-01A1 (MB), P30- NS04758 (CBSCR), Craig H. Neilsen Foundation 316721 (JG), a Florence P. Kendall Post-Professional Doctoral Scholarship (TF) and a Promotion of Doctoral Studies—Level I Scholarship from the Foundation for Physical Therapy (TF).

## Conflict of Interest Statement

The authors declare that the research was conducted in the absence of any commercial or financial relationships that could be construed as a potential conflict of interest.

## Abbreviations

BBB: Basso, Beattie, Bresnahan Locomotor Rating Scale open field locomotor test
ILP: instrumental learning paradigm
SCI: spinal cord injury
TX: transection of spinal cord.
